# Pediatric heart-kidney transplantation

**DOI:** 10.1016/j.jhlto.2025.100393

**Published:** 2025-09-24

**Authors:** Swati Choudhry, Kriti Puri, Vikas R. Dharnidharka

**Affiliations:** a Department of Pediatrics, Section of Pediatric Cardiology, Texas Children’s Hospital, Baylor College of Medicine, Houston, TX; b Department of Pediatrics, Division of Pediatric Nephrology, Rutgers Robert Wood Johnson Medical School, Bristol-Myers Squibb Children's Hospital, New Brunswick, NJ

**Keywords:** Heart kidney transplantation, Heart transplantation, Simultaneous heart kidney transplantation

## Abstract

Heart transplantation is regarded as the definitive treatment for advanced pediatric heart failure. However, concomitant kidney dysfunction often complicates candidate selection for isolated heart transplantation. Prolonged venous congestion, nephrotoxic exposures, and recurrent episodes of acute kidney injury can result in varying degrees of renal impairment. Differentiating between reversible kidney injury secondary to cardiorenal syndrome and irreversible intrinsic renal disease remains a major challenge. Pediatric heart-kidney transplantation has shown survival benefits for patients with end-stage heart failure requiring chronic dialysis, yet its role in patients with less severe renal dysfunction is less clearly defined. This review summarizes the current evidence on patient selection, perioperative and post-transplant management, and ethical considerations for pediatric heart-kidney transplantation.

## Background

The field of pediatric heart transplantation has experienced remarkable progress since its inception in the late 1960′s.^1^ Extensive evidence indicates that patients with advanced heart failure awaiting heart transplantation have long-standing central venous congestion, leading to variable degrees of kidney dysfunction that can be worsened by a low cardiac output state, also known as cardiorenal syndrome.^2^, ^3^, ^4^ Patients with a failing Fontan circulation listed for heart transplantation present unique challenges that predispose them to a rapid decline in renal function. These challenges include prior bypass surgeries, persistent venous hypertension, low cardiac output state, and chronic hypoxemia.^4^ Distinguishing between patients with reversible kidney injury due to cardiorenal syndrome, who may recover kidney function after heart transplantation, and those with intrinsic advanced kidney disease, who would benefit most from combined heart-kidney transplant, is challenging.^2^, ^3^, ^4^

The number of patients undergoing combined heart and kidney transplantation has increased significantly in recent years.^2^, ^3^, ^4^, ^5^ In fact, the kidney is the most frequently transplanted extrathoracic organ combined with heart transplantation, accounting for over three-quarters of multi-organ transplants.^3^ Most of the data available for combined heart-kidney transplantation are from adult studies,^2^, ^3^, ^4^, ^5^, ^6^ with a few published studies in pediatrics.^4^, ^7^, ^8^, ^9^ A national pediatric cohort study demonstrated that end-stage renal disease (ESRD) adversely impacts the survival of heart transplant recipients.^7^ However, advanced heart failure patients with ESRD who undergo kidney transplantation after heart transplantation experience improved survival and superior quality of life compared to those on dialysis.^7^, ^8^, ^9^ The benefits of heart-kidney transplantation for patients on chronic dialysis are becoming increasingly evident.^2^, ^3^, ^4^, ^5^, ^6^, ^7^, ^8^, ^9^ However, determining the appropriate timing for dual-organ transplantation in patients with less severe renal disease remains unclear.^2^, ^3^

This review offers an evidence-based framework for candidate selection and outcomes in pediatric combined heart–kidney transplantation, highlighting the unique challenges of dual-organ transplantation and the critical role of multidisciplinary collaboration. Given the limited pediatric data, we highlight pediatric-specific considerations wherever possible while incorporating relevant adult-based challenges with combined heart-kidney transplantation that may have implications for the pediatric population.

### Types of heart kidney transplantation

A combined heart-kidney transplant can be categorized as: a) a simultaneous heart-kidney transplant (SHKTx, with both organs from the same donor) or b) a sequential heart-kidney transplant (two organs from separate donors).^4^ The rise in simultaneous heart-kidney (SHKTx) transplants is largely due to the current allocation system, which prioritizes SHKTx candidates over those waiting for kidney transplants alone (KTx), despite the absence of standardized criteria for SHKTx eligibility.^2^, ^3^ This creates an incentive to favor SHKTx over heart transplant alone (HTx) when the reversibility of kidney dysfunction in HTx candidates is uncertain. In contrast, patients with persistent kidney dysfunction needing renal replacement therapy following heart transplantation do not receive such prioritization and must face standard wait times for a deceased donor kidney unless a living donor is available.^2^, ^3^ These policies raise ethical concerns, as SHKTx candidates often receive priority over KTx candidates who may have been waiting longer and could derive equal or greater benefits from transplantation. Establishing selection criteria for pediatric combined organ transplant recipients is essential to ensure fair and optimal utilization of scarce donor resources.^4^

### Indications

Combined heart-kidney transplants are rare in pediatric patients, and the absence of clear selection criteria makes the practice highly center-dependent.^4^ The 2006 adult guidelines from the International Society for Heart and Lung Transplantation recommended that irreversible renal dysfunction, defined by an estimated glomerular filtration rate (eGFR) of <40 mL/min/m², be considered a relative contraindication for heart transplantation alone.^10^ Evidence indicates that SHKTx confers a survival advantage compared with HTx alone in patients with chronic dialysis dependence.^9^ Karamlou et al, in a national adult cohort, identified an eGFR threshold <37 mL/min/1.73 m² at which combined transplantation should be recommended.^5^ Similarly, in a national pediatric cohort, simultaneous heart-kidney transplantation was associated with significantly improved survival compared with isolated heart transplantation among patients with an eGFR ≤35 mL/min/1.73 m².^9^

In 2019, the American Society for Transplantation hosted a consensus conference to establish standards for simultaneous heart-kidney transplant eligibility.^2^ The Heart Kidney work group recommended nephrologist evaluation for SHKTx should begin when glomerular filtration rate (GFR) is <45 mL/min/1.73 m². They proposed that patients with eGFR <30 mL/min/1.73 m² may be considered for SHKTx, those with eGFR > 45 mL/min/1.73 m² were likely inappropriate, and those with eGFR 30-44 mL/min/1.73 m² and clear indicators of chronic kidney disease, such as small kidney size or persistent proteinuria >0.5 g/day, could also be considered on an individual basis. These guidelines were not enforced by UNOS, leaving decisions to individual centers.^2^

Despite evidence showing limited benefit for SHKTx in patients with eGFR > 40 mL/min/1.73 m², significant variability existed, raising concerns about resource allocation fairness.^2^, ^3^ The 2023 UNOS policy introduced stricter SHKTx criteria, limiting kidney allocation to HTx candidates with severe CKD or sustained AKI.^11^ This policy permits programs to proceed with isolated heart transplantation while allowing time for renal recovery. Candidates whose kidney function fails to improve may qualify for safety net priority, which grants HTx recipients expedited access to kidney transplantation within one year if they remain dialysis-dependent or have a sustained eGFR ≤20 mL/min/1.73 m² for at least six weeks between 30 and 365 days post HTx.^2^, ^11^ The safety net is designed to mitigate premature kidney listing and promote isolated HTx when renal recovery remains possible.

### Surgery

Heart-kidney transplantation can be performed using two primary techniques. The first approach is a "simultaneous" heart-kidney transplant, where the kidney transplant is performed either simultaneously with the heart transplant as a single operation, where the kidney is implanted immediately after the heart transplant, or after several hours of stabilization, utilizing organs from the same deceased donor as a staged procedure with a brief delay before kidney implantation.^4^ The second method is "sequential" heart-kidney transplantation, in which the heart transplant is performed first, followed by the kidney transplant after a delay of days, months, or even years, often using a kidney from a different donor.^4^, ^12^

There are no standardized guidelines for selecting one approach over the other, as each has distinct advantages and limitations and may be driven by the patient's perioperative clinical status and hemodynamic stability. The staged method is generally favored for a simultaneous heart-kidney transplant, as it allows for hemodynamic stabilization between the two transplants.^4^, ^12^ The staged approach helps mitigate risks associated with inflammatory response during cardiopulmonary bypass, and perioperative hemodynamic instability, which can adversely affect kidney allograft, function. Heart-kidney transplants from a single donor may promote immune tolerance, potentially reducing the incidence of cardiac allograft rejection and coronary allograft vasculopathy.^13^ On the contrary, Ruzza and colleagues have reported that the sequential approach may be superior.^14^ They noted that the inflammatory surge and hemodynamic instability associated with cardiopulmonary bypass during the simultaneous heart-kidney approach could negatively impact the transplanted kidney in the immediate perioperative period.^14^ The sequential approach allows patients to recover from heart transplant surgery before undergoing a kidney transplant. However, it comes with challenges, including prolonged ischemic allograft time and increased immunogenicity of the renal parenchyma, which elevate the risk of rejection and reduce long-term survival.^4^, ^12^

### Outcomes

A recent national transplant database study by Itagaki et al, involving 1124 simultaneous heart-kidney transplant cases, reported superior survival in heart-kidney transplant recipients compared to heart transplantation alone for both dialysis-dependent recipients and non-dialysis-dependent recipients with a GFR of up to approximately 40 mL/min/1.73 m² reaffirming the notion outlined in the 2006 adult guidelines from the International Society for Heart and Lung Transplantation.^3^, ^10^ ESRD significantly increases mortality risk after pediatric heart transplantation, with a 2- to 6-fold higher risk of death compared to those without ESRD.^7^, ^8^ A nationwide retrospective cohort study found that pediatric heart transplant recipients who remained on chronic dialysis until death or last follow-up had a 31 times higher hazard of death than those who received kidney transplants (HR, 31.4; 95% CI, 21.0-48.4; *P* < 0.0001).^7^

In a pediatric case series by Weng et al, the mean age of simultaneous heart-kidney transplant recipients was 13 years, with a 5-year survival rate of 85.7% after SHKTx, statistically similar to the survival after pediatric HTx alone.^15^ There is emerging data that combined heart-kidney transplantation is a promising option for patients with end-stage heart failure with severe kidney dysfunction, offering survival rates comparable to those of isolated heart or kidney transplants.^2^, ^3^ Findings from adult studies have shown that heart re-transplant recipients with ESRD have improved survival with combined heart and kidney transplants than with heart re-transplantation alone.^16^ In a national outcomes study, Karamlou et al found that adult heart transplant recipients with a pre-transplant eGFR below 37 mL/min have worse survival than those who undergo combined heart-kidney transplantation.^5^ A national database study on simultaneous pediatric heart-kidney transplant recipients suggested that pediatric heart-kidney transplantation should be considered for heart transplant candidates with kidney failure requiring dialysis or an eGFR below 35 mL/min.^9^ In another study using data from the United Network for Organ Sharing (UNOS), Gill et al reported a significant improvement in survival for dialysis-dependent patients who received combined heart-kidney transplants, compared to those who had isolated heart transplants.^6^ There was no survival difference between heart-kidney transplant recipients and those not on dialysis at the time of transplant. However, combined heart-kidney transplants showed a lower 1-year survival rate when compared to kidney transplants alone from deceased donors (84.0% vs. 94.5%, *P* < 0.001).^6^

In a retrospective cohort study using a national transplant database, Itagaki et al examined outcomes of simultaneous heart-kidney transplantation, focusing on post-transplant kidney allograft failure and loss.^3^ They found that while simultaneous heart-kidney transplantation offered better survival than heart transplantation alone for both dialysis-dependent and non-dialysis-dependent recipients with GFRs up to approximately 40 mL/min/1.73 m², it nearly doubled the risk of kidney allograft loss compared to recipients of contralateral isolated kidney allografts.^3^ There is an ambiguity in identifying patients who would benefit most from simultaneous heart-kidney transplantation versus heart transplant alone, as kidney function may recover after isolated heart transplantation restores heart function. Unlike the well-defined criteria for isolated heart transplantation, the guidelines for simultaneous heart-kidney transplantation remain unclear. As such, careful candidate selection is warranted to maximize kidney allocation, particularly for heart transplant patients who are not on dialysis.^2^, ^3^, ^4^ Due to the scarcity of donor organs, it is crucial for the transplant community to ensure that allocating a donor kidney for combined transplantation is justified, especially when it might have a significant impact if used in the kidney transplant pool.^2^, ^3^

### Ventricular assist device

Data on SHKTx in patients supported by durable LVADs is limited. Several studies, including Ruzza et al, Schaffer et al, and Zalawadiya et al reported comparable survival rates between SHKT recipients with or without MCS.^14^, ^17^, ^18^ Similarly, Melehy et al also reported no significant survival difference between patients with and without pre-transplant MCS in the SHKTx subgroup, though those requiring extracorporeal membrane oxygenation had worse outcomes.^19^ These studies included both temporary and durable MCS. In a national database study by Zalawadiya et al, pre-transplant dialysis—not MCS status—was the primary determinant of post-SHKTx dialysis requirements, which emerged as a significant risk factor for both in-hospital and long-term mortality.^17^

Contrary to the above findings, Russo et al reported that mechanical circulatory support use was associated with poor survival among SHKTx recipients.^20^ Similarly, Atkin's study showed that patients with durable LVADs undergoing SHKTx had worse short- and long-term survival and higher rates of post-transplant dialysis.^21^ While the reasons for these differences remain unclear, Atkins et al outlined several potential explanations.^21^ First, patients with LVAD explants may experience greater hemodynamic instability and vasoplegia post-transplant, requiring higher doses of inotropic and vasopressor support. These hemodynamic disturbances may affect the transplanted kidney more severely than the native kidney, contributing to higher post-transplant dialysis rates. Additionally, patients with LVADs may experience more severe coagulopathy, potentially due to acquired von Willebrand syndrome, which can complicate surgery and recovery. Additionally, elevated bilirubin levels in the SHKTx group, compared to heart transplant alone recipients, suggest increased liver congestion or dysfunction, which may contribute to perioperative bleeding, hypotension, and post-transplant right-heart failure, ultimately affecting renal graft recovery.

### Delayed graft function

Delayed graft function is defined as the need for dialysis within the first 7 days following kidney transplantation.^22^ It is a known complication after multi-organ transplantation and can adversely affect graft outcomes. The average incidence of delayed graft function is approximately 30% in kidneys transplanted from deceased donors.^23^ In a national database study of over 1100 simultaneous heart-kidney transplant recipients, Parajuli et al reported that pre-transplant dialysis was a significant risk factor and was associated with an almost fourfold higher risk for K-DGF than not being on dialysis before transplant.^23^

### Immunosuppression

Induction therapy regimens and practices vary widely among institutions. There is currently no consensus regarding the use of induction therapy in SHKTx. When used, the choice of agent should be tailored to the recipient's risk factors for infection, delayed graft function, or immunologic profile. For recipients at risk of delayed graft function, anti-thymocyte globulin may be considered.^2^ In contrast, basiliximab is preferred for those with lower immunologic or infection risks.^2^ Delaying the initiation of calcineurin inhibitors (CNI) for kidney protection is generally unnecessary; however, a short delay may be warranted with anti-thymocyte globulin if kidney function is impaired postoperatively. If basiliximab is used, CNIs should be introduced within 24 hours. The Heart/Kidney Workgroup also suggested that corticosteroids may be weaned for a select group of low-risk recipients, defined as having no treated rejection episodes, no donor-specific antibodies, and normal heart and kidney function.^2^ Maintenance immunosuppression after heart transplantation typically involves a combination of a CNI, an antimetabolite agent, and a corticosteroid. Tacrolimus is preferred over cyclosporine as the CNI due to its lower incidence of rejection, nephrotoxicity, hypertension, hyperlipidemia, and diabetes. Mycophenolate mofetil is the preferred antimetabolite agent.

Sirolimus, a mammalian target of rapamycin (mTOR) inhibitor, is used to reduce acute rejection and prevent cardiac allograft vasculopathy. However, it is not initiated immediately after transplantation due to its potential to exacerbate the nephrotoxic effects of CNIs and impair wound healing. Sirolimus is typically introduced 3-6 months post-transplant, aiming to slow kidney dysfunction progression and improve kidney function over the following year. Studies have shown that combining sirolimus with CNIs produces synergistic immunosuppression, as the two drugs target sequential steps in immune activation. Using sirolimus with reduced doses of CNIs has been shown to slow kidney dysfunction progression compared to full-dose CNI therapy. Another potential option to reduce CNI nephrotoxicity in SHKTx includes using belatacept, a selective T-cell costimulation blocker. Although the U.S. Food and Drug Administration has not approved belatacept for heart transplant recipients, it may be considered an alternative immunosuppressive therapy in the future to help mitigate CNI-related nephrotoxicity in SHKTx recipients.^24^

Several studies have shown that simultaneous heart and kidney transplantation results in significantly lower rejection rates for both the heart and kidney allografts compared to transplanting each organ separately. While the exact mechanisms remain unknown, data suggests that combined organ transplantation may promote tolerance or reduce the need for immunosuppressive therapy.

## Conclusion

Kidney dysfunction portends worse outcomes both in patients awaiting heart transplantation and after heart transplant, with the worst impact for patients requiring dialysis. Overall, combined organ transplantation is a reasonable option for candidates with end-stage heart and kidney failure, with outcomes comparable to those of isolated heart transplant recipients, signifying that the added surgery does not considerably increase the perioperative risk. In 2023, UNOS implemented strict criteria for SHKTx listing with a safety net policy to prioritize kidney transplantation for those HTx recipients with severe kidney dysfunction early after HTx to maximize scarce donor resources.^11^

There is a definite advantage in performing simultaneous heart-kidney transplant over heart transplantation alone in chronic dialysis-dependent patients. Patients with an established GFR of less than 30 mL/min/1.73 m² who are not dialysis-dependent may be eligible for SHKTx. A possible benefit for select non–dialysis-dependent recipients with an established GFR between 30 and 44 mL/min/1.73 m² who have firm evidence of CKD—such as small kidney size or persistent proteinuria more than 0.5 g/day in stable hemodynamics—may also qualify for SHKTx on a case-by-case basis. Patients with a GFR of 45-59 mL/min/1.73 m² are generally not considered suitable candidates for SHKTx. However, they may benefit from a proposed safety net policy for kidney transplantation after heart transplantation wherein HTx alone recipients would receive priority for kidneys if they experience chronic dialysis dependence or maintain a persistent GFR of 20 mL/min/1.73 m² or lower for at least 6 weeks between days 30 and 365 post-transplant.

Glomerular filtration rate estimates based on creatinine-based formulas overestimate measured kidney function in heart transplant recipients.^4^ This is particularly evident in patients with chronic illness and low muscle mass, which results in lower serum creatinine levels. Cystatin C-based glomerular filtration rate estimates are more reliable. It is essential to develop uniform pediatric criteria for heart-kidney transplantation given the scarce donor organ supply to maximize the benefit of organ allocation. The effectiveness of the new SHKTx policy and safety net outcomes for HTx recipients with severe kidney dysfunction remains to be evaluated.

## Declaration of Competing Interest

The authors declare that they have no known competing financial interests or personal relationships that could have appeared to influence the work reported in this paper.

## References

[1] Dipchand A.I., Kirk R., Edwards L.B. (2013). The Registry of the International Society for heart and lung transplantation: Sixteenth Official Pediatric Heart Transplantation Report--2013; focus theme: age. J Heart Lung Transplant.

[2] Kobashigawa J., Dadhania D.M., Farr M. (2021). Consensus conference on heart-kidney transplantation. Am J Transplant.

[3] Itagaki S., Toyoda N., Moss N. (2023). Outcomes of simultaneous heart and kidney transplantation. J Am Coll Cardiol.

[4] Choudhry S., Denfield S.W., Dreyer J.W. (2019).

[5] Karamlou T., Welke K.F., McMullan D.M. (2014). Combined heart-kidney transplant im-proves post-transplant survival compared with isolated heart transplant in recipients with reduced glomerular filtration rate: analysis of 593 combined heart- kidney transplants from the united network organ sharing database. J Thorac Cardiovasc Surg.

[6] Gill J., Shah T., Hristea I. (2009). Outcomes of simultaneous heart-kidney transplant in the US: a retrospective analysis using OPTN/UNOS data. Am J Transpl.

[7] Choudhry S., Dharnidharka V.R., Castleberry C.D. (2018). End-stage renal disease after pediatric heart transplantation: a 25-year national cohort study. J Heart Lung Transpl.

[8] Ruebner R.L., Reese P.P., Denburg M.R. (2013). End-stage kidney disease after pediatric non-renal solid organ transplantation. Pediatrics.

[9] Choudhry S., Denfield S.W., Dharnidharka V.R. (2022). Simultaneous pediatric heart-kidney transplant outcomes in the US: a-25 year national cohort study. Pediatr Transpl.

[10] Mehra M.R., Kobashigawa J., Starling R. (2006). Listing criteria for heart transplantation: International Society for Heart and Lung Transplantation guidelines for the care of cardiac transplant candidates—2006. J Heart Lung Transpl.

[11] Swanner K, Schmitt L, Messick E. Establish eligibility criteria and safety net for heart-kidney and lung-kidney allocation. 2023. (This source delineates the updated organ allocation policy for heart-kidney transplantation).

[12] Jain R., M Kittleson M.K. (2024). Evolutions in combined heart-kidney transplant. Curr Heart Failure Rep.

[13] Narula J., Bennett L.E., DiSalvo T., Hosenpud J.D., Semigran M.J., Dec G.W. (1997). Outcomes in re-cipients of combined heart-kidney transplantation: multiorgan, same-donor trans-plant study of the International Society of Heart and Lung Transplantation/united network for organ sharing scientific registry. Transplantation.

[14] Ruzza A., Czer L.S.C., Trento A., Esmailian F. (2013). Combined heart and kidney transplantation: What is the appropriate surgical sequence?. Interact CardioVascular Thoracic Surg.

[15] Weng P.L., Alejos J.C., Halnon N., Zhang Q., Reed E.F., Tsai Chambers E. (2017). Long-term outcomes of simultaneous heart and kidney transplantation in pediatric recipients. Pediatr Transpl.

[16] Savla J., Lin K.Y., Pradhan M. (2015). Heart retransplant recipients have better survival with concurrent kidney transplant than with heart retransplant alone. J Am Heart Assoc.

[17] Zalawadiya S.K. (2016). Mechanical circulatory support and simultaneous heart-kidney transplantation: an outcome analysis. J Heart Lung Transpl.

[18] Schaffer J.M., Chiu P., Singh S.K., Oyer P.E., Reitz B.A., Mallidi H.R. (2014). Heart and combined heartkidney transplantation in patients with concomitant renal insufficiency and end-stage heart failure. Am J Transpl.

[19] Melehy A., Sanchez J.E., Nemeth S.K. (2023). National outcomes of bridge to multiorgan cardiac transplantation using mechanical circulatory support. J Thoracic Cardiovasc Surg.

[20] Russo M.J., Rana A., Chen J.M. (2009). Pretransplantation patient characteristics and survival following combined heart and kidney transplantation: an analysis of the united network for organ sharing database. Arch Surg.

[21] Atkins J., Nicholas H.R., Sheng J.R. (2022). Outcomes in patients with LVADs undergoing simultaneous heart-kidney transplantation. J Cardiac Failure.

[22] Yarlagadda S.G., Coca S.G., Garg A.X. (2008). Marked variation in the definition and diagnosis of delayed graft function: a systematic review. Nephrol Dial Transpl.

[23] Parajuli S., Karmin A., Muth B. (2021). Risk factors and outcomes for delayed kidney graft function in simultaneous heart and kidney transplant recipients: a UNOS/OPTN database analysis. Am J Transpl.

[24] Kittleson M.M., Sharma K., Brennan D.C. (2023). Dual-organ transplantation: indications, evaluation, and outcomes for heart-kidney and heart-liver transplantation: a scientific statement from the American Heart Association. Circulation (New York, N. Y. ).

